# A case of necrotizing pneumonia leading to respiratory failure and tracheostomy

**DOI:** 10.1016/j.idcr.2025.e02480

**Published:** 2026-01-06

**Authors:** Nurul 'Aifaa Mohd Azmi, Shao Keong Koeh, Tuan Sharifah Syuwaibah Tuan Zainal, Mohd Zulfakar Mazlan

**Affiliations:** a Department of Anaesthesiology and Intensive Care, School of Medical Sciences, Universiti Sains Malaysia, 16150, Kota Bharu, Kelantan, Malaysia; b Department of Anaesthesiology and Intensive Care, Hospital Pakar Universiti Sains Malaysia, 16150 Kota Bharu, Kelantan, Malaysia

**Keywords:** Necrotizing pneumonia, Meropenem, Linezolid, Intensive care unit

## Abstract

Necrotizing pneumonia is a severe lung infection characterized by pulmonary necrosis and high mortality rates of up to 50 %. It is typically caused by toxin-producing pathogens, such as *Staphylococcus aureus* and *Klebsiella pneumoniae*. Limited guidelines exist for its management, making its treatment challenging. Case report: We discuss a case of a 58-year-old Malay woman with no significant comorbidity but developed necrotizing pneumonia. The diagnosis was confirmed using computed tomography (CT) of the thorax. Treatment with carbapenem, oxazolidinone and corticosteroid led to significant recovery. Conclusion: To date, no specific anti-inflammatory treatments exist for severe necrotizing pneumonia. Since systemic inflammation and multi-organ failure drive mortality, management focuses on supportive care aimed at maintaining oxygenation and hemodynamic stability to improve outcomes in critically ill patients.

## Introduction

Necrotizing pneumonia is a life-threatening form of severe pneumonia characterized by pulmonary necrosis and often associated with severe sepsis. Necrotizing pneumonia has a reported incidence of 1 % of pneumonia with a mortality rate of up to 50 % [1]. The causative organisms are usually toxin-producing, such as *Staphylococcus aureus, Streptococcus pneumoniae, and Klebsiella pneumoniae*. To date, there are limited guidelines for the management of necrotizing pneumonia, and its treatment is mainly based on severe pneumonia treatment. The postulated pathogenesis is severe lung parenchymal infection triggering a systemic inflammatory response, leading to tissue necrosis and liquefaction of infected tissue. Its complications are pulmonary tissue destruction, thrombus formation, and empyema.

## Case report

A 58-year-old Malay woman with a history of well-controlled childhood asthma, working as a schoolteacher, presented to the emergency department with a 5-day history of fever, chills, rigor, and productive cough. She had no known history of COVID-19 infection. On day 2 of her illness, she visited an outpatient clinic where hypotension was noted, but she declined hospital admission. Her condition subsequently worsened with increasing breathlessness, prompting presentation to our facility.

**Fig. 1:**
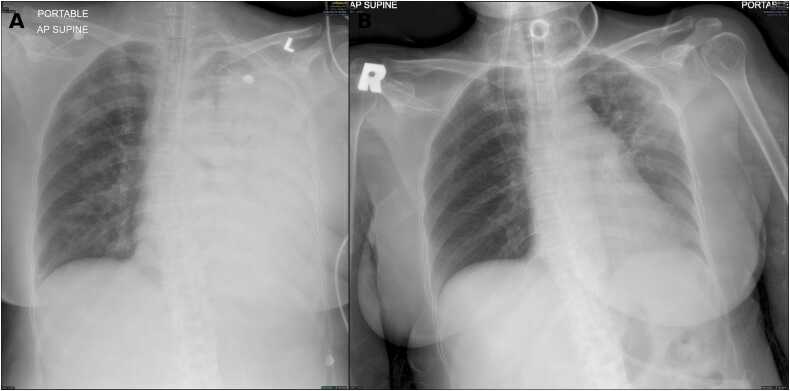
Portable anteroposterior chest radiographs obtained during hospitalisation. (a) Chest radiograph on Day 3 of admission demonstrating near-complete opacification of the left hemithorax. (b) Chest radiograph on Day 50 showing marked interval radiographic improvement with re-expansion of the left lung.

On arrival, she was conscious but tachypneic with a respiratory rate of 39 breaths per minute and oxygen saturation of 83 % on room air. Her blood pressure was 120/56 mmHg, and heart rate was 115 beats per minute. Physical examination revealed lethargy, fever, and coarse crepitations over the left lung; other systemic examinations were unremarkable. SARS-CoV-2 polymerase chain reaction testing performed on admission was negative. Arterial blood gas analysis showed severe hypoxic respiratory failure with lactic acidosis. Laboratory investigations revealed hemoglobin 14.6 g/dL, white cell count 8.7 × 10^9/L (neutrophils 91 %, lymphocytes 7.6 %), and platelets 168 × 10^9/L. Initial chest radiograph demonstrated heterogeneous opacities in the left lower and upper lung fields and right perihilar region (Fig. 1(a)). Electrocardiogram was unremarkable.

She was diagnosed with severe pneumonia and started on high-flow nasal cannula oxygen therapy (fraction of inspired oxygen (FiO_2_) 0.9, 60 L/min) and fluid resuscitation (20 mL/kg over 2 h). Empiric intravenous antibiotics included intravenous ceftazidime 2 g stat and azithromycin 500 mg once daily. Blood cultures were obtained. Despite treatment, repeated arterial blood gases showed persistent hypoxia with a partial pressure of oxygen in arterial blood (PaO_2_)/FiO_2_ ratio (P/F) of 58. Due to respiratory distress and failure to improve on high-flow oxygen, she was intubated. Intubation was uneventful, but ventilation was difficult, with a P/F ratio of 100 and a downward trend in blood pressure requiring norepinephrine infusion. She was admitted to the intensive care unit (ICU).

**Fig. 2:**
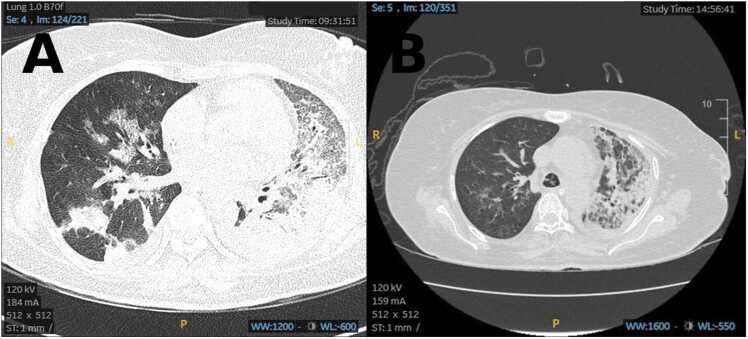
Axial computed tomography images of the thorax obtained during hospitalisation. (A) CT scan on Day 5 of admission demonstrating extensive bilateral parenchymal opacities with predominant left-sided involvement. (B) CT scan on Day 26 showing interval radiological improvement with partial resolution of parenchymal changes.

In the ICU, she required high ventilator support, with a P/F ratio improving to 160. Repeat chest X-ray on day 2 admission showed worsening and persistent pneumonic infiltrates on the left lung (Fig. 1(b)). Antibiotics were escalated to intravenous levofloxacin and cloxacillin to cover possible *Staphylococcus infection*. Bronchoscopy on days 2 and 3 revealed minimal mucus plugs and mucosal erythema; bronchial alveolar lavage cultures were negative. Nasal swabs for respiratory viral panels were also negative. Given chest radiographs showed progressive consolidation in the left lung, prompting contrast-enhanced CT thorax on day 4 of illness, which demonstrated severe necrotizing pneumonia with radiological features suggestive of secondary organizing pneumonia and acute respiratory distress syndrome changes in the left upper and lower lobes (Fig. 2(a)).

During this period, blood investigations reflected a severe systemic inflammatory response with involvement of multiple organ systems, including markedly raised inflammatory markers (peak C-reactive protein 550 mg/L and procalcitonin 17.51 ng/mL), worsening acute kidney injury with serum creatinine rising to 325 µmol/L, and deranged liver biochemistry with elevated bilirubin and alkaline phosphatase levels (Table 1, Table 2).

**Table 1:** Timeline of clinical events during hospitalisation. Chronological summary of major clinical events and interventions during the course of illness and hospitalisation.

**Day of Admission**	**Event Summary**
Day −5	Onset of fever, chills, rigor, productive cough
Day −2	Outpatient visit: hypotension noted; patient declined admission
Day 0 (Admission)	Presented to Emergency Department (ED) with severe respiratory distress; diagnosed with severe pneumonia; started High Flow Nasal Canula (HFNC) oxygen and IV antibiotics (ceftazidime, azithromycin); blood cultures taken; intubated due to worsening hypoxia and respiratory failure; admitted to Intensive Care Unit (ICU)
Day 1–2	Bronchoscopy performed; minimal mucus plugs; lavage bacterial cultures negative; respiratory viral panel negative. Antibiotics were escalated to intravenous levofloxacin and cloxacillin to cover possible *Staphylococcus infection*.
Day 4	CT thorax showed severe necrotizing pneumonia with radiological features suggestive of organizing pneumonia
Day 10	Repeat bronchoscopy and cultures negative; deterioration noted
Day 10–19	IV dexamethasone 6 mg daily administered
Day 10–23	Antibiotics escalated to IV linezolid and meropenem
Day 21	Tracheostomy performed due to prolonged ventilation
Day 24	Repeat in-hospital CT thorax showed resolution of necrotizing pneumonia
Day 32	Successfully weaned off mechanical ventilation
Day 40	Transfer to medical HDU ward
Day 43	Blood culture grew *Klebsiella Pneumonia* and *Enterococcus Faecium*
Day 57	Discharge home

**Table 2:** Serial laboratory investigations during hospitalisation. Serial laboratory investigations recorded during hospitalisation, demonstrating changes in haematological, inflammatory, renal, and liver parameters over time.

**A Haematological parameters**				
**Day**	**WCC**	**PLT**	**Hb**	**HCT**
Day 1	10.79	101	11.2	34.6
Day 2	15.02	105	11.7	36.3
Day 3	13.98	95	10.8	33.2
Day 5	12.04	214	10.3	30.5
Day 8	14.81	355	9.9	29.9
Day 10	9.72	413	7.2	21.8
Day 15	10.79	366	10.2	30.4
Day 25	9.15	166	10.0	30.9
Day 45	11.3	321	8.4	26.1

B Inflammatory markers		
**Day**	**CRP**	**PCT**
Day 1	550	—
Day 2	471	—
Day 3	—	17.51
Day 5	—	—
Day 8	—	—
Day 10	140	5.99
Day 15	66.4	—
Day 25	28.7	—
Day 45	3.78	—

C Renal function		
**Day**	**Urea**	**Creatinine**
Day 1	12.1	133
Day 2	14.6	150
Day 3	17.8	165
Day 5	33.3	262
Day 8	33.8	246
Day 10	31.2	293
Day 15	27.5	325
Day 25	19.3	129
Day 45	12.0	52

D Liver function					
**Day**	**ALT**	**AST**	**ALP**	**TB**	**ALB**
Day 1	79	131	194	33	21
Day 2	80	136	212	34	24
Day 3	68	120	246	51	25
Day 5	72	126	272	50	29
Day 8	53	57	276	52	24
Day 10	42	59	321	34	22
Day 15	37	58	569	12	25
Day 25	23	28	362	9	30
Day 45	21	22	161	4	33

Abbreviations: WCC, white cell count; PLT, platelet count; Hb, haemoglobin; HCT, haematocrit; CRP, C-reactive protein; PCT, procalcitonin; ALT, alanine aminotransferase; AST, aspartate aminotransferase; ALP, alkaline phosphatase; TB, total bilirubin; ALB, albumin. Missing values indicate tests not performed on that day.

Due to clinical deterioration and worsening chest x-ray, bronchoscopy was repeated on day 10; cultures and polymerase chain reaction (PCR) for bacterial and viral pathogens remained negative. She received intravenous dexamethasone 6 mg daily for 10 days and antibiotic escalation to intravenous linezolid 600 mg twice daily and meropenem 1 g three times daily for two weeks. Subsequently, ventilator support gradually weaned pressure support by day 20. However, renal function worsened during ICU stay, necessitating regular dialysis (Table 2).

Given prolonged ventilation, tracheostomy was performed on day 21. Repeat in-hospital CT thorax was performed on day 24 (Fig. 2(b)) after completing antibiotics, showed resolution of necrotizing pneumonia. She was successfully liberated from mechanical ventilation by day 32 and transferred to the high dependency unit (HDU) on day 40 with tracheostomy oxygen mask at 15 L/min and discharged home on day 50 with marked improvement on chest radiograph (Fig. 1(b)). In HDU, her blood culture grew *Klebsiella pneumoniae* and *Enterococcus faecium* which is sensitive to cefuroxime and vancomycin and treated with those antibiotics prior to discharge.

## Discussion

Necrotizing pneumonia is a rare yet life-threatening spectrum of severe pneumonia. This diagnosis is rather unexpected in this patient, considering her healthy premorbid function as a teacher with no significant comorbidity. Commonly quoted risk factors include alcohol use, and diabetes mellitus [1]. However, none of these risk factors were present in our patient upon presentation. β-lactams and macrolides were initially prescribed in accordance with international guidelines in the treatment of severe pneumonia [2], [3]. Its poor clinical and radiographic response despite antibiotic and bronchoscopic lavage leads to suspicion of malignancy, interstitial lung disease, and lung abscess as differential diagnoses. CT of the thorax was subsequently done on day 4 of illness and unveiled this rather unexpected diagnosis of necrotizing pneumonia. CT thorax imaging typically reveals disruption of the normal lung structure accompanied by areas of reduced contrast uptake in the lung tissue, indicating liquefactive necrosis [1], [4]. These findings reflect impaired perfusion and tissue destruction characteristic of necrotizing pneumonia. Chest X-ray imaging was insufficient to confirm necrotizing pneumonia due to its low sensitivity and specificity [1].

β-lactams and macrolides are first-line treatments as recommended by the latest guidelines to empirically treat *Streptococcus pneumoniae, Klebsiella pneumoniae*, *Staphylococcus*, and atypical organisms [2]. The CT thorax findings and poor clinical response during the first week of treatment raised suspicion of poor response with the levofloxacin and cloxacillin. Although no microbiological isolation of methicillin-resistant *Staphylococcus aureus* (MRSA) was identified, the radiological pattern of bilateral cavitating and necrotizing infiltrates, together with the lack of clinical response to levofloxacin and subsequent marked clinical, biochemical, and radiographic improvement following initiation of linezolid, strongly suggests MRSA as the most likely causative pathogen. Resistance to fluoroquinolones is well described in MRSA pneumonia, making poor response to levofloxacin plausible. Intravenous meropenem and linezolid were therefore empirically started in this patient for two weeks.

Following that, she had significant clinical, biochemical, and radiographic improvement. Linezolid was chosen over vancomycin for two reasons: it is effective at neutralizing toxin [5], [6], [7] produced by MRSA and does not require regular therapeutic monitoring (TDM) [8], especially vital since the patient had acute renal failure requiring dialysis.

To date, there is no optimal duration of antibiotic treatment recommended for severe necrotizing pneumonia [4]. However, C-reactive protein (CRP) and procalcitonin (PCT) are often recommended to guide antibiotic duration [9], [10], [11]. As for this patient, her CRP and point of care PCT were decreased after 2 weeks of meropenem and linezolid. Thus, we decided to stop antibiotics in view of the good clinical and biochemical response. Although PCT couldn't discriminate between viral and bacterial infection, the higher titer suggests an increased likelihood of bacterial pneumonia. Furthermore, PCT is well studied to guide the duration of antibiotics [12], [13], potentially shortening the antibiotic course, especially in such patients with life-threatening necrotizing pneumonia. Studies show antibiotic duration guided by PCT associated with a reduction of 1–2 days of treatment [10], [11], [14]. In contrast, *Klebsiella pneumoniae* isolated later in the clinical course was most consistent with a secondary nosocomial infection, likely related to prolonged intensive care unit stay and invasive devices, rather than the primary cause of the initial severe necrotizing pneumonia.

Radiological features suggestive of secondary organizing pneumonia were noted on her contrast-enhanced CT thorax. Histopathologically, organizing pneumonia is characterized by patchy filling of the alveoli and bronchioles with plugs of granulation tissue [15]. On CT imaging, it most commonly appears as bibasal, peribronchovascular, and/or peripheral areas of consolidation [16]. However, these radiological features may overlap with changes seen in the acute phase of severe pneumonia, and a definitive diagnosis requires histopathological confirmation or interval follow-up imaging [15]. In this patient, tissue biopsy was not performed as she responded well to broad-spectrum antibiotics and corticosteroid treatment, and follow-up CT imaging at 3–6 months after discharge was not available as the patient did not return for follow-up.

Corticosteroid treatment remains controversial. The Dexamethasone for the acute respiratory distress syndrome (DEXA-ARDS) trial (2020) demonstrated that early administration of dexamethasone significantly reduced the duration of mechanical ventilation and ICU mortality in patients with moderate to severe ARDS [17]. In contrast, the Early Steroid Administration in Community-Acquired Pneumonia with High Inflammatory Response (ESCAPE) trial, which involved 586 patients, found no difference in ICU mortality when using methylprednisolone at 0.75 mg/kg/day for 10 days [18].

Our center used IV dexamethasone 6 mg OD for 10 days for this patient after weighing the risk of immunosuppression, potential secondary infection, and previous experience in treating COVID-related organizing pneumonia. This dose regimen is also like the Randomised Evaluation of COVID-19 Therapy (RECOVERY) trial, which used IV dexamethasone in COVID-19 pneumonia [19]. Given the apparent clinical response to corticosteroid therapy, systemic vasculitis was also considered as a differential diagnosis. However, the absence of extrapulmonary manifestations, normal renal parameters and urinalysis, and sustained clinical recovery without relapse on follow-up make active systemic vasculitis unlikely in this patient.

The patient showed excellent clinical and biochemical improvement following broad-spectrum empirical antibiotics and a short course of intravenous dexamethasone. At follow-up reviews at 3 and 6 months after discharge, she remained clinically well with no constitutional symptoms, skin lesions, haematuria, neurological deficits, or other features suggestive of systemic vasculitis. Therefore, it is important to adhere to pneumonia guidelines to improve patient outcomes [20]. Early surgical referrals are paramount for the timely detection and management of complications such as empyema and bronchopleural fistula [21].

## Conclusion

This case highlights the rarity and complexity of severe necrotizing pneumonia in a patient without clear predisposing risk factors. Radiological features suggestive of organizing pneumonia were observed during the disease course; however, these findings should be interpreted with caution given the overlap with acute and subacute pneumonia. Accurate assessment using advanced imaging such as CT thorax remains vital in guiding management. Tailored empirical antibiotic therapy, guided by biomarkers including C-reactive protein and procalcitonin, together with judicious use of corticosteroids, contributed to a favourable clinical and radiological outcome. Further studies are needed to better define optimal antimicrobial strategies, treatment duration, and the role of corticosteroids in severe necrotizing pneumonia.

## Author contributions

ZF, KH, SS, and FA contributed to the analysis and interpretation of the patient data for this case and played major roles in drafting the manuscript. They were also involved in the conception of the study and in critically revising the manuscript. All authors reviewed and approved the final version of the manuscript.

## Declarations

Ethics approval and consent to participate are not applicable.

## CRediT authorship contribution statement

**Shao Keong Koeh:** Writing – original draft. **Mohd Azmi Nurul 'aifaa:** Writing – review & editing, Supervision, Resources. **Mohd Zulfakar Mazlan:** Writing – review & editing, Visualization, Supervision, Methodology, Formal analysis, Data curation, Conceptualization. **Tuan Sharifah Syuwaibah Tuan Zainal:** Writing – review & editing.

## Consent

Written informed consent was obtained from the next of kin for publication of this case report and accompanying images during ICU admission. A copy of the written consent is available for review by the Editor-in-Chief of this journal on request.

## Consent for publication

Consent for publication was not required because the study did not involve any identifiable or personal data.

## Ethical approval

Not required for Case report in my institution.

## Funding

This work was supported by 10.13039/501100004595Universiti Sains Malaysia external grant R504‑LR‑GAL008‑0006150268‑N109 and short-term grant R501-LR-RND002-0000001210-0000.

## Declaration of Competing Interest

All authors have no conflicts of interest.

## Data Availability

All data in the medical record and will be available if requested.
